# Relationships between human serum albumin levels and septic shock, in-hospital, and out-of-hospital mortality in elderly patients with pneumonia in different BMI ranges

**DOI:** 10.1186/s41479-024-00138-8

**Published:** 2024-09-25

**Authors:** Sha Huang, Lanlan Chen, Ning Yang, Jiao Zhang, Yan Wang, Xiaoyan Chen

**Affiliations:** Zigong Affiliated Hospital of Southwest Medical University, Department of Geriatric, Zigong, China

**Keywords:** Human serum albumin (HSA), Body mass index (BMI), Pneumonia, Septic shock, Mortality

## Abstract

**Objective:**

This retrospective cohort identified the association of human serum albumin (HSA) with adverse outcomes (septic shock, in-hospital and out-of-hospital mortality) in elderly hospitalized patients who have community-acquired pneumonia (CAP) and specific body mass index (BMI).

**Materials and methods:**

This research included hospitalized CAP individuals (≥ 60 years) and was conducted at a teaching hospital in western China. All the patients were categorized into three populations based on two BMI cutoff values (18.5 kg/m^2^ and 24 kg/m^2^). The data was acquired from medical records, local government mortality databases, and telephone interviews. Binomial logistic regression analysis was used to explore the associations between low HSA and septic shock and in-hospital mortality, and Cox regression analysis was used to explore the association between low HSA and out-of-hospital mortality.

**Results:**

A total of 627 patients were included in the analysis of in-hospital death and septic shock, and 431 patients were included in the analysis of out-of-hospital death. The study showed that 120 elderly patients with CAP (19.14%) died in the hospital, while 141 patients (32.71%) died out of the hospital, and 93 patients (14.83%) developed septic shock. No differences in in-hospital and out-of-hospital mortality were observed for BMI values < 18.5 kg/m^2^ or BMI ≥ 24 kg/m^2^, regardless of whether HSA was ≥ 40 g/l or < 40 g/l. When 18.5 kg/m^2^ ≤ BMI < 24 kg/m^2^, patients with HSA < 40 g/l had both higher in-hospital and out-of-hospital mortality compared with those with HSA ≥ 40 g/l (in-hospital death: 26.13% vs. 11.46%, *p* < 0.001; out-of-hospital death: 46.15% vs. 19.17%, *p* < 0.001). No significant differences were observed in the incidence of septic shock between patients with HSA < 40 g/l and those with HSA ≥ 40 g/l either in the overall population or when the BMI values were divided according to the cutoff values of 18.5 kg/m^2^ and 24 kg/m^2^. After further logistic regression analysis and adjustment for potential confounders, the results showed that when 18.5 kg/m^2^ ≤ BMI < 24 kg/m^2^, elderly CAP patients with HSA < 40 g/l had a higher risk of in-hospital and out-of-hospital mortality compared with those with HSA ≥ 40 g/l (in-hospital death: HR = 1.964, 95%CI = 1.08–3.573; out-of-hospital death: HR = 2.841, 95%CI = 1.745–4.627).

**Conclusions:**

HSA levels can predict the risk of in-hospital and out-of-hospital mortality in elderly patients with CAP and normal BMI values. However, HSA cannot predict the risk of septic shock in elderly patients hospitalized with CAP, irrespective of their BMI classification.

**Supplementary Information:**

The online version contains supplementary material available at 10.1186/s41479-024-00138-8.

## Introduction

Elderly people are prone to community-acquired pneumonia (CAP) [1, 2], and septic shock is a fatal complication of severe CAP [3]. Myint et al. found that the mortality rate of CAP patients was 28.42% [4]. Elderly CAP patients are more than twice the risk of death than younger patients [5]. One study showed that the median cost of outpatient treatment for elderly patients with CAP was $346, while the median cost of hospitalization was $4,851 [6]. The more severe the condition, the higher the cost of treatment in the hospital [6]. The economic and clinical burden of elderly CAP patients is very heavy [7, 8] and is not paid enough attention to.

Human serum albumin (HSA) is a negatively charged, multifunctional plasma protein, primarily synthesized in the liver, and accounts for > 50% of the whole plasma protein content [9]. HSA mainly exists in the reduced form with free thiols [9] and a half-life of about 20 days [10]. The literature suggests that it is associated with poor prognosis in various diseases. For example, He et al. pointed out that low HSA was independently associated with the risk of severe CAP (individuals who require invasive mechanical ventilation and vasopressor for septic shock) in pregnancy [11]. Lim et al. found that low HSA was an independent factor responsible for CAP patients’ death [12]. Low HSA on admission predicts death among acutely ill hospitalized patients [13]. Rudasill et al. also found that low preoperative HSA predicts septic shock and death after a laparoscopic cholecystectomy [14].

Nelson et al. separately investigated the association of HSA and body mass index (BMI) with post-total knee replacement complications (including septic shock) and death [15]. BMI is a recognized biomarker of malnutrition [16], and according to multiple research, it is associated with poor clinical prognosis. Low BMI is specifically linked with high mortality risk in middle-aged and elderly (40–79 years) CAP patients [17], septic shock [18], and severe COVID-19 [19]. In addition, it is also related to the risk of septic shock in critically ill COVID-19 and pregnant CAP patients [11, 19]. High BMI is associated with both disease recurrence and mortality in patients with breast cancer [20] and with mortality risk in patients with cardiogenic shock [21]. COVID-19 patients with BMI ≥ 40 kg/m^2^ had significantly increased all-cause in-hospital mortality, requirements for invasive mechanical ventilation and its associated mortality, as well as the incidence of septic shock [22].

The possible mechanism by which HSA can predict death is that, on the one hand, the HSA level reflects the nutritional status of the body, and on the other hand, HSA levels are also affected by inflammation and infection [16, 23]. Therefore, the present study aimed to investigate the use of HSA for predicting mortality risk in patients with CAP in terms of their BMI values. This is, to a certain extent, equivalent to considering the nutritional status of the patients. It was hypothesized that the HSA levels measured under these conditions may be a better reflection of the influence of inflammation and infection. In addition, a study of elderly hospitalized patients showed that their mortality rate during hospitalization was 26%, while the mortality increased to 44% during the 12-month follow-up period [24]. This reminds us that the risk of death among the elderly after discharge from the hospital is also worthy of attention.

There has been no previous exploration of the association between HSA levels and the risk of in-hospital and out-of-hospital mortality in elderly patients with CAP in relation to their BMI. Therefore, the present study aimed to answer the following questions: 1. Does low HSA increase the risk of septic shock and death (in-hospital and out-of-hospital) in elderly patients with CAP and low BMI? 2. Does low HSA increase the risk of septic shock and death (in-hospital and out-of-hospital) in people with normal BMI? 3. Does low HSA increase the risk of septic shock and death (in-hospital and out-of-hospital) in people with high BMI?

## Study methodology

### Design of the study and patient demographics

This observational research was conducted retrospectively at a teaching hospital in western China from January 2016 to March 2021. Hospitalized CAP individuals aged 60 years and above were included, and those with limb edema, missing BMI, or HSA were excluded. Patients who had been lost to follow-up were excluded from the analysis of out-of-hospital deaths. In addition, patients who had died in the hospital were excluded from the analysis of out-of-hospital deaths.

### Ethics

All data were anonymized, and the study was overseen by the Center for Health Informatics. The confidentiality of the data was upheld throughout. The investigation followed the Declaration of Helsinki principle and was authorized by the Research Ethics Committee of Zigong Affiliated Hospital of Southwest Medical University, Zigong Mental Health Center (No. 2021–06-01). As it is retrospective research, the Research Ethics Committee waived the requirement for informed consent.

### Data collection

After admission, the patient's clinical information was collected, including a history of drinking and smoking, chronic conditions, sex, age, height, weight, and blood test results. Dementia, stroke history, chronic obstructive pulmonary disease (COPD), diabetes, coronary heart disease (CHD), and hypertension are chronic diseases [25]. The cutoff value of HSA was 40 g/ L; that is, HSA < 40 g/ L was considered low, and HSA ≥ 40 g/ L was considered high [14, 26]. BMI was computed by dividing weight by the square of height (kg/m^2^). The cut-off value for BMI was 18.5 kg/m^2^ and 24 kg/m^2^, where < 18.5 kg/m^2^ = low BMI populations, 18.5 kg/m^2^ ≤ BMI < 24 kg/m^2^ = normal BMI populations, and BMI ≥ 24 kg/m^2^ = high BMI populations [27]. Septic shock was recorded as one of the outcome indicators. For septic shock, the diagnostic criteria by Font et al. in 2020 were followed [28]. Information on deaths was retrieved from medical records or local government databases, and telephone interviews were conducted in case of the unavailability of this data.

### Statistical analysis

The SPSS 25.0 software was utilized for statistical measurements. Two-sided p-values < 0.05 was deemed a significance threshold. Normally distributed continuous variables were presented as mean ± SD and other data as median (quartile). The Student's t-test or Rank-sum tests were utilized for constructing baseline features. For the statistical measurement of categorical variables, data were presented as numbers (percentages), and Pearson's chi-square test was applied for comparing baseline characteristics. For older patients with CAP, binomial logistic regression analysis was used to explore the relationship between HSA and septic shock and in-hospital mortality, and Cox regression analysis was used to explore the relationship between HSA and out-of-hospital mortality. In addition, the population was divided into groups according to BMI (BMI < 18.5 kg/m [2], 18.5 kg/m^2^ ≤ BMI < 24 kg/m^2^, BMI ≥ 24 kg/m^2^), and used logistic regression and Cox regression to explore their relationships with in-hospital and out-of-hospital mortality. Two models were developed. Model 1 was uncorrected, and Model 2 was corrected (in single-factor analysis, variables with *P* < 0.05 are included in the study).

## Results

**Fig. 1:**
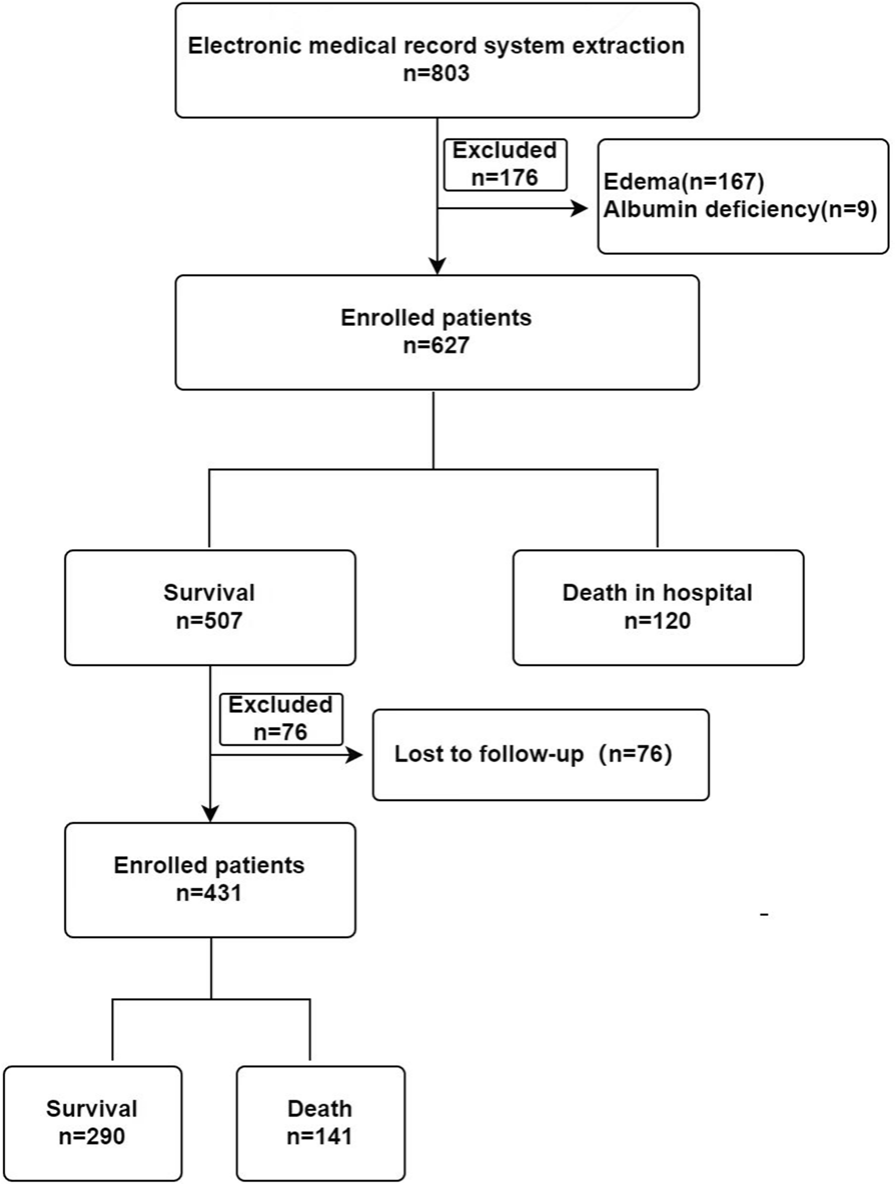
Flowchart of patient inclusion and exclusion

A total of 627 patients were included in the analysis of in-hospital death and septic shock, and 431 patients were included in the analysis of out-of-hospital death (see Fig. 1). The study showed that 120 elderly CAP patients (19.14%) died in the hospital, while 141 patients (32.71%) died out of hospital, and 93 patients (14.83%) developed septic shock. In the analysis of in-hospital deaths, the low, normal, and high BMI populations were 72 (11.48%), 467 (74.48%), and 88 (14.04%), respectively. In the analysis of out-of-hospital deaths, the low, normal, and high BMI populations were 46 (10.67%), 315 (73.09%), and 70 (16.24%), respectively. There were statistically significant differences in terms of age, sex, COPD, and septic shock between patients who died and survived in the hospital (Table 1). There were no statistically significant differences in smoking history, drinking history, diabetes, hypertension, CHD, stroke history, and dementia (Table 1). Significant differences were observed in terms of age, CHD, and dementia between patients who died and those who survived out of hospital (Table 2), while no significant differences were seen in sex, smoking history, drinking history, diabetes, hypertension, COPD, history of stroke, and septic shock (Table 2).

**Table 1:** Baseline characteristics of participants according to in hospital death

Characteristics	Survival *N* = 507	In hospital death *N* = 120	P-value
**Age, years, median(iqr)**	78(68,85)	75.25(82.5,88.75)	** < 0.001**
**Sex, n (%)**			**0.017**
male	304(77.95)	86(22.05)	
female	203(85.65)	34(14.35)	
**Smoking history, n (%)**			0.474
no	297(79.84)	75(20.16)	
yes	207(82.14)	45(17.86)	
**Drinking history, n (%)**			0.96
no	370(80.79)	88(19.21)	
yes	133(80.61)	32(19.39)	
**Diabetes, n (%)**			0.176
no	416(81.89)	92(18.11)	
yes	91(76.47)	28(23.53)	
**Hypertension, n (%)**			0.522
no	305(80.05)	76(19.95)	
yes	202(82.11)	44(17.89)	
**CHD, n (%)**			0.834
no	364(81.07)	85(18.93)	
yes	143(80.34)	35(19.66)	
**COPD, n (%)**			**0.047**
no	384(79.18)	101(20.82)	
yes	123(86.62)	19(13.38)	
**Stroke history, n (%)**			0.087
no	293(83.24)	59(16.76)	
yes	214(77.82)	61(22.18)	
**Dementia, n (%)**			0.177
no	389(82.07)	85(17.93)	
yes	118(77.12)	35(22.88)	
**Septic shock, n (%)**			** < 0.001**
no	464(86.89)	70(13.11)	
yes	43(46.24)	50(53.76)	

*Note*: *CHD *coronary heart disease, *COPD *chronic obstructive pulmonary disease

**Table 2:** Baseline characteristics of participants according to the out of hospital death

Characteristics	No death (out of hospital) *N* = 290	Death (out of hospital) *N* = 141	P-value
**Age, years, median(iqr)**	75.5(67, 83)	83(76, 87.5)	** < 0.001**
**Sex, n (%)**			0.102
male	159(64.11)	89(35.89)	
female	131(71.58)	52(28.42)	
**Smoking history, n (%)**			0.736
no	180(68.7)	82(31.3)	
yes	108(65.06)	58(34.94)	
**Drinking history, n (%)**			0.925
no	218(67.49)	105(32.51)	
yes	69(66.35)	35(33.65)	
**Diabetes, n (%)**			0.967
no	237(67.33)	115(32.67)	
yes	53(67.09)	26(32.91)	
**Hypertension, n (%)**			0.434
no	178(68.73)	81(31.27)	
yes	112(65.12)	60(34.88)	
**CHD, n (%)**			**0.001**
no	216(72.24)	83(27.76)	
yes	74(56.06)	58(43.94)	
**COPD, n (%)**			0.297
no	227(68.58)	104(31.42)	
yes	63(63)	37(37)	
**Stroke history, n (%)**			0.121
no	171(70.37)	72(29.63)	
yes	119(63.3)	69(36.7)	
**Dementia, n (%)**			** < 0.001**
no	231(72.64)	87(27.36)	
yes	59(52.21)	54(47.79)	
**Septic shock, n (%)**			> 0.99
no	289(67.37)	140(32.63)	
yes	1(50)	1(50)	

*Note*: *CHD *coronary heart disease, *COPD *chronic obstructive pulmonary disease

Bold: variable is statistically significant between the two groups

At BMI values < 18.5 kg/m^2^, there were no significant differences in in-hospital and out-of-hospital mortality, regardless of HSA levels ≥ 40 g/l or < 40 g/l (Table 3). When 18.5 kg/m^2^ ≤ BMI < 24 kg/m^2^, patients with HSA < 40 g/l showed both higher in-hospital and out-of-hospital mortality than those with HSA ≥ 40 g/l (in-hospital mortality: 26.13% vs. 11.46%, *p* < 0.001; out-of-hospital mortality: 46.15% vs. 19.17%, *p* < 0.001; Table 3). At BMI values ≥ 24 kg/m^2^, there was no difference in in-hospital and out-of-hospital mortality, regardless of whether HSA was ≥ 40 g/l or < 40 g/l (Table 3). On analysis of the overall population or after the division of the BMI into two cutoff values of 18.5 kg/m^2^ and 24 kg/m^2^, no significant difference was found in the incidence of septic shock between patients with HSA < 40 g/l and HSA ≥ 40 g/l (Table S1).

**Table 3:** Differences in HSA between death and survival groups

Variable	Survival (In hospital *n* = 507)	Death (In hospital *n* = 120)	P-value	Survival (Out of hospital *n* = 290)	Death (Out of hospital *n* = 141)	P-value
**BMI < 18.5 kg/m**^**2**^**, n (%)**			0.218			0.266
HSA ≥ 40 g/l	18(90)	2(10)		11(78.57)	3(21.43)	
HSA < 40 g/l	38(73.08)	14(26.92)		18(56.25)	14(43.75)	
**18.5 kg/m**^**2**^** ≤ BMI < 24 kg/m**^**2**^**, n (%)**			** < 0.001**			** < 0.001**
HSA ≥ 40 g/l	139(88.54)	18(11.46)		97(80.83)	23(19.17)	
HSA < 40 g/l	229(73.87)	81(26.13)		105(53.85)	90(46.15)	
**BMI ≥ 24 kg/m**^**2**^**, n (%)**			0.63			0.195
HSA ≥ 40 g/l	51(96.23)	2(3.77)		37(90.24)	4(9.76)	
HSA < 40 g/l	32(91.43)	3(8.57)		22(75.86)	7(24.14)	

*Note*: *BMI *body mass index, *HSA *human serum albumin

Bold: variable is statistically significant between the two groups

Further logistic regression showed that when 18.5 kg/m^2^ ≤ BMI < 24 kg/m^2^, patients with HSA < 40 g/l had a higher risk of both in-hospital and out-of-hospital death than those with HSA ≥ 40 g/l (in-hospital death: OR = 2.731, 95%CI = 1.572–4.746; out-of-hospital death: HR = 3.553, 95%CI = 2.237–5.643; Table 4). After adjusting for potential confounding factors, the results still showed that compared with elderly CAP patients with HSA ≥ 40 g/l, patients with HSA < 40 g/l had a higher risk of both in-hospital and out-of-hospital mortality (in-hospital mortality: OR = 1.964, 95% CI = 1.08–3.573; out-of-hospital mortality: HR = 2.841, 95% CI = 1.745–4.627; Table 4). In addition, there was no association between normal BMI and either in-hospital or out-of-hospital mortality compared with BMI < 18.5kg/m^2^ (Table S2).

**Table 4:** Correlations between HSA and death

Variable	Model 1		Model 2	
	**P-value**	**HR/OR (95% *CI*)**	**P-value**	**HR/OR (95% *CI*)**
**In hospital death**				
**18.5 kg/m**^**2**^** ≤ BMI < 24 kg/m**^**2**^				
HSA ≥ 40 g/l	-	1	-	1
HSA < 40 g/l	< 0.001	2.731(1.572–4.746)	0.027	1.964(1.08–3.573)
**Out of hospital death**				
**18.5 kg/m**^**2**^** ≤ BMI < 24 kg/m**^**2**^				
HSA ≥ 40 g/l	-	1	-	1
HSA < 40 g/l	< 0.001	3.553(2.237–5.643)	< 0.001	2.841(1.745–4.627)

Note:

Model 1: a non-adjusted model

Model2: adjusting for age, sex, COPD, septic shock in hospital death; adjusting for age, CHD, dementia in the out of hospital death

*BMI *body mass index, *HSA *human serum albumin, *CHD *coronary heart disease, *COPD *chronic obstructive pulmonary disease

## Discussion

This study used BMI cutoff values to distinguish different populations and found that HSA levels could only predict in-hospital and out-of-hospital mortality in elderly patients with CAP who had normal BMI. The innovation of this study is that it is the first time that the BMI cutoff value has been used to distinguish the population and to explore the relationship between low HSA levels and septic shock, in-hospital and out-of-hospital mortality in elderly patients with CAP and different BMI values. This has clinical application value. In patients with normal BMI, the HSA level can be used to assess the risk of in-hospital and out-of-hospital mortality in elderly patients with CAP. It is recommended that clinical staff be aware that HSA levels should not be ignored for patients with a normal BMI range, while for patients with septic shock, other indicators should be determined to assess the risk of occurrence. This is meaningful as a theoretical basis for medical communication with patients and their families. HSA is inexpensive, has a long half-life, and can be comprehensively measured in medical institutions [10]. In addition, the findings also showed that the out-of-hospital mortality rate (32.71%) of elderly patients with CAP was higher, suggesting that clinicians need to pay attention to the prognosis of these patients after discharge.

The inflammatory mechanisms involved are: 1) HSA is produced in the liver and is suppressed in acute situations, which increases the release of positive acute phase proteins, such as C-reactive protein, and the higher the levels of this protein are, the worse the prognosis [29–32]. 2) The intravascular and extravascular HSA distribution is altered in critical illness, and the altered distribution is associated with increased capillary leakage caused by bacterial endotoxins, cytokines (TNF-α and IL-6), chemokines, etc. [31, 33]. 3) HSA has transport functions (fatty acids and drugs) and may indirectly affect inflammatory pathways and microvascular integrity [9, 31] because HSA has various physiological functions in multiple organs; therefore, after severe infection, HSA drops sharply, and the homeostasis of the body is severely disrupted.

The literature has indicated that HSA predicts the risk of death without distinguishing BMI ranges [34–38]. Since the majority of the studies were based on patients with a normal BMI, overall results might be affected. The reason why HSA cannot predict septic shock remains unclear. The adjusted factors included age, sex, COPD, CHD, septic shock, and dementia, all of which have been associated with mortality risk in elderly patients with CAP in published studies [39–43].

This survey showed that in the analysis of in-hospital and out-of-hospital deaths, the proportions of elderly patients with CAP in the low, normal, and high BMI populations were 11.48/10.67%, 74.48/73.09%, and 14.04/16.24%, respectively. Li et al. revealed that the proportions of low, normal, and high BMI populations were 7.44%, 57.18%, and 35.38% in patients undergoing open gastrointestinal surgery [27]. The difference in these two studies might be because of different study populations (hospitalized elderly CAP patients vs. patients undergoing open gastrointestinal surgery) and large age difference in the study subjects (the median age of subjects in this study was 79 years, and the average age in Li et al.'s study was 55.17–58.06 years old) [27].

This investigation showed that 14.83% of elderly patients with CAP developed septic shock. Sellarès-Nadal et al. showed in their research that the incidence of septic shock in CAP patients was 10.3% [44]. The reasons for the difference mainly include age and the proportion of smoking patients [44, 45]. Specifically, the median and mean age of the subjects was 79 and 65 years, and the proportion of smokers was 40.38% and 22% in this investigation and in the study by Sellarès-Nadal et al., respectively [44]. Welte et al. found that the mortality rate of hospitalized CAP patients ranged from 5 to 20% [41], whereas that in our study was 19.14%.

The limitations of this investigation were. 1) this was retrospective research conducted in only one hospital, so there may be selection bias. Prospective studies with more subjects should be conducted to verify this conclusion. 2) due to the small study size, the population was only divided by two cut-off values of BMI. Furthermore, the investigation also lacked a finer division of elderly CAP patients based on more cut-off values of HSA. 3) due to the small study size, the classification analysis on deaths (such as short- and long-term death, etc.) was not performed. 4) The cause of death is highly useful for assessing confounding factors or completely different causes of death unrelated to CAP, but since the information on death was mainly determined through telephone follow-up, it was possible that the patient's family did not have this precise information. In addition, the customs and medical conditions of the region result in the performance of almost no autopsies for patients, so the cause of death was difficult to determine. Therefore, there was no follow-up and further analysis of the cause of death.

## Conclusions

HSA levels can predict the risk of in-hospital and out-of-hospital mortality in elderly patients with CAP and normal BMI but cannot predict the risk of in-hospital and out-of-hospital mortality in patients with either low or high BMI. In addition, the HSA level was unable to predict the risk of septic shock in elderly patients hospitalized with CAP, irrespective of their BMI.

## Supplementary Information

Supplementary Material 1.

Supplementary Material 2.

## Data Availability

The datasets generated and analyzed during the current study are not publicly available, as this is a database containing a lot of important information on which we are working on several important projects, but they are now also available from the corresponding author on reasonable request.

## Abbreviations

HSA: Human serum albumin
CAP: Community-acquired pneumonia
BMI: Body mass index
COPD: Chronic obstructive pulmonary disease
CHD: Coronary heart disease
