# Supplementary material for: Relationships between human serum albumin levels and septic shock, in-hospital, and out-of-hospital mortality in elderly patients with pneumonia in different BMI ranges
Source: Pneumonia (Nathan). 2024 Sep 25;16:17. doi: 10.1186/s41479-024-00138-8 (PMC11423505; doi:10.1186/s41479-024-00138-8)
Supplement: Supplementary file 1 — Supplementary Material 1. [file 41479_2024_138_MOESM1_ESM.docx]

**Table S1 Differences in HSA between** **septic shock and non-septic shock groups**

| **Variable** | **Non-septic shock**  **(n=534)** | **Septic shock**  **(n=93)** | **P-value** |
| --- | --- | --- | --- |
| **Total, n (%)** |  |  | 0.233 |
| HSA≥40g/l | 201(87.39) | 29(12.61) |  |
| HSA<40g/l | 333(83.88) | 64(16.12) |  |
| **BMI<18.5kg/m^2^, n (%)** |  |  | 0.447 |
| HSA≥40g/l | 18(90) | 2(10) |  |
| HSA<40g/l | 41(78.85) | 11(21.15) |  |
| **18.5kg/m^2^ ≤BMI<24kg/m^2^ ,n (%)** |  |  | 0.427 |
| HSA≥40g/l | 137(87.26) | 20(12.74) |  |
| HSA<40g/l | 262(84.52) | 48(15.48) |  |
| **BMI≥24kg/m^2^ ,n (%)** |  |  | 0.885 |
| HSA≥40g/l | 46(86.79) | 7(13.21) |  |
| HSA<40g/l | 30(85.71) | 5(14.29) |  |

**Note:** BMI: body mass index; HSA: human serum albumin.
