# Supplementary material for: Relationships between human serum albumin levels and septic shock, in-hospital, and out-of-hospital mortality in elderly patients with pneumonia in different BMI ranges
Source: Pneumonia (Nathan). 2024 Sep 25;16:17. doi: 10.1186/s41479-024-00138-8 (PMC11423505; doi:10.1186/s41479-024-00138-8)
Supplement: Supplementary file 2 — Supplementary Material 2. [file 41479_2024_138_MOESM2_ESM.docx]

**Table S2 Correlations between BMI and death**

| **Variable** | **Model 1** | | **Model 2** | |
| --- | --- | --- | --- | --- |
|  | **P-value** | **HR/OR (95% *CI*)** | **P-value** | **HR/OR (95% *CI*)** |
| **In hospital death** | | | | |
| BMI<18.5kg/m^2^ | - | 1 | - | 1 |
| 18.5kg/m^2^ ≤BMI<24kg/m^2^ | 0.891 | 0.966(0.587-1.589) | 0.444 | 0.783(0.418-1.466) |
| BMI≥24kg/m^2^ | <0.001 | 0.263(0.129-0.536) | 0.009 | 0.238(0.081-0.701) |
| **Out of hospital death** | | | | |
| BMI<18.5kg/m^2^ | - | 1 | - | 1 |
| 18.5kg/m^2^ ≤BMI<24kg/m^2^ | 0.943 | 1.019(0.612-1.697) | 0.369 | 0.789(0.196-0.903) |
| BMI≥24kg/m^2^ | 0.01 | 0.368(0.172-0.787) | 0.026 | 0.42(1.019-1.06) |

Note:

Model 1: a non-adjusted model.

Model2: adjusting for age, sex, COPD, septic shock, in hospital death.

adjusting for age, CHD, dementia in the out of hospital death.

**Note:** BMI: body mass index; CHD: coronary heart disease; COPD: chronic obstructive pulmonary disease.
